# Impact of body fat content on extracorporeal shock wave lithotripsy for pancreatic duct stones: a retrospective cohort study

**DOI:** 10.7717/peerj.21112

**Published:** 2026-04-09

**Authors:** WeiChen Ning, Yan Shi, Yadi Ju, Yihai Shi

**Affiliations:** 1 School of Gongli Hospital Medical Technology, University of Shanghai for Science and Technology, Shanghai, China; 2 Pudong New Area Gongli Hospital, Shanghai, Pudong New Area, China

**Keywords:** Visceral fat, Pancreatic duct stones, Computed tomography, Extracorporeal shock wave lithotripsy, Obesity, Body fat percentage

## Abstract

**Background:**

The impact of body fat content on the treatment efficacy of extracorporeal shock wave lithotripsy (ESWL) for pancreatic duct stones (PDS) remains unclear, and there is a paucity of targeted analyses on refined body composition parameters in existing studies. This study aims to investigate the associations between body mass index (BMI), body fat percentage, visceral adipose tissue/skeletal muscle tissue (VAT/SMT) ratio, and the treatment efficiency of ESWL for PDS.

**Methods:**

This was a single-center retrospective observational study that enrolled 147 patients with PDS who underwent ESWL and achieved complete stone clearance at Gongli Hospital, Pudong New Area, Shanghai, from January 2021 to January 2025. Measurements of VAT, subcutaneous adipose tissue (SAT), and SMT at the level of the L3/L4 intervertebral space were performed using non-contrast computed tomography (CT) combined with Adobe Photoshop software, and the VAT/SMT ratio was calculated accordingly. Hierarchical stepwise linear regression models were employed to analyze the independent effects of body fat-related parameters (including BMI, body fat percentage, and VAT/SMT ratio) on lithotripsy time and the number of lithotripsy sessions, after adjusting for confounding variables such as gender, age, and stone cross-sectional area.

**Results:**

Pearson correlation analysis revealed that body fat percentage exhibited a strong positive correlation with the total lithotripsy time (*r* = 0.544, *P* < 0.001) and the number of lithotripsy sessions (*r* = 0.546, *P* < 0.001). Meanwhile, BMI (*r* = 0.354∼0.382, *P* < 0.001) and the VAT/SMT ratio (*r* = 0.352tsim0.353, *P* < 0.001) showed a moderate positive correlation with both outcomes. Multivariate linear regression analysis (fully adjusted model) confirmed that body fat percentage (number of lithotripsy sessions: β = 0.38, *P* < 0.001; total lithotripsy time: β = 0.37, *P* < 0.001), BMI (number of lithotripsy sessions: β = 0.27, *P* < 0.001; total lithotripsy time: β = 0.32, *P* < 0.001), and the VAT/SMT ratio (number of lithotripsy sessions: β = 0.20, *P* = 0.017; total lithotripsy time: β = 0.18, *P* = 0.035) were all independent positive predictors of lithotripsy efficiency. Additionally, stone cross-sectional area also significantly influenced the outcomes (number of lithotripsy sessions: β = 0.23, *P* = 0.001; total lithotripsy time: β = 0.15, *P* = 0.027).

**Conclusion:**

BMI, body fat percentage, and VAT/SMT ratio exhibit a significant positive correlation with the total lithotripsy time and number of lithotripsy sessions for ESWL in PDS. Among these parameters, body fat percentage has the strongest association with lithotripsy efficiency and maintains a stable independent predictive effect in multivariate models.

**Trial registration:**

Clinical study registration number: GLYY1s2025-032.

## Background

Chronic pancreatitis (CP) is characterized by irreversible pancreatic fibrosis, and pancreatic duct stones (PDS) are one of its most common complications, with an incidence exceeding 50% at the time of CP diagnosis (Beyer et al., 2020; Thierens et al., 2025; Hines & Pandol, 2024). These stones directly induce intractable abdominal pain and pancreatic exocrine/endocrine failure by causing pancreatic duct obstruction, parenchymal ischemia, and increased intraductal pressure; thus, stone clearance is the core therapeutic goal for alleviating CP symptoms (Gerges, Beyna & Neuhaus, 2023; Tandan, Pal & Reddy, 2023). Epidemiological studies have shown that approximately 70%–90% of patients with PDS have a history of CP, among which alcohol exposure is recognized as a key etiological factor (Beyer et al., 2022).

Extracorporeal shock wave lithotripsy (ESWL) has emerged as the first-line treatment for main pancreatic duct stones larger than 5 mm. By focusing high-energy shock waves to fragment stones, it creates favorable conditions for subsequent endoscopic stone extraction, with a complete stone clearance rate of 69.8%–74.3% (Van Huijgevoort et al., 2020). Unlike ESWL for renal calculi, ESWL for PDS demands higher positioning accuracy and energy control (Yi, Xu & Hu, 2024; Tandan & Reddy, 2011). The pancreas is located retroperitoneally, adjacent to vital organs, and there are inherent differences in composition between PDS and urinary calculi. High-density stones require higher energy for effective fragmentation; however, the pancreas has low energy tolerance, creating a therapeutic dilemma (Takahashi et al., 1975). Specialized equipment is therefore needed to adjust shock wave parameters to avoid pancreatic injury (Abdul, Jiao & Wu, 2025; Patel & Gómez, 2020).

ESWL is a commonly used non-invasive method for treating urinary calculi (Malinaric et al., 2023; Manzoor, Leslie & Saikali, 2024), but its efficacy is influenced by multiple factors (Duan et al., 2023; Abdul, Jiao & Wu, 2025). In addition to stone-related factors, individual patient factors also exert a certain impact (Abdul, Jiao & Wu, 2025; Wagenius et al., 2022). In obese patients, the thick fat layer causes significant shock wave energy attenuation, impairing stone positioning and fragmentation efficacy (Snicorius et al., 2021). We observed that factors such as subcutaneous fat thickness, visceral fat accumulation, and body fat percentage have not been investigated in terms of their impact on ESWL efficiency for PDS. Therefore, exploring the influence of these factors on lithotripsy efficiency holds clinical value for improving treatment efficacy and minimizing the number of lithotripsy sessions for patients.

Existing studies have confirmed that ESWL efficacy is influenced by factors such as stone diameter and computed tomography (CT) attenuation value (Duan et al., 2023; Abdul, Jiao & Wu, 2025), but the role of individual patient factors remains unclear. In the field of renal calculi, obesity has been shown to reduce ESWL efficiency through two mechanisms: first, the acoustic impedance difference between adipose tissue and normal tissue causes shock wave energy attenuation. Adipose tissue has significantly lower acoustic impedance than muscle tissue, leading to energy reflection and attenuation of shock waves at the fat-muscle interface (Moghimnezhad, Shahidian & Andayesh, 2021). Second, the thick fat layer interferes with the coupling between the transducer and the skin, reducing stone positioning accuracy. This manifests as blurred stone images during ESWL, making it difficult to precisely lock the focal point and resulting in the failure of shock wave energy to be accurately transmitted to the stones (Allard et al., 2012; Kim et al., 2015; Kim et al., 2014). However, the impact of body fat parameters on ESWL for PDS remains understudied. Existing literature either only focuses on the association between body mass index (BMI) and CP progression or is limited to analyzing the impact of fat on renal calculi ESWL, with no investigations into the role of refined parameters such as visceral adipose tissue (VAT) and subcutaneous adipose tissue (SAT) in PDS lithotripsy efficiency. For instance, recent studies have found that sarcopenic obesity-related parameters exhibit greater predictive value in the pathogenesis of CP (Yoon et al., 2017; Inci et al., 2012). More importantly, traditional indicators such as BMI cannot distinguish between visceral and subcutaneous obesity (Ji et al., 2019), and VAT has been shown to have a significantly stronger association with CP severity than BMI (Angadi et al., 2024; Kumar et al., 2017). The clinical value of these refined body composition parameters thus urgently needs to be explored.

From a physical mechanism perspective, the deep anatomical location of PDS may amplify the interfering effect of adipose tissue: the fat pad formed by VAT surrounding the pancreas increases the shock wave transmission distance (Kumar et al., 2017; Lozova et al., 2025), while SAT directly impairs the focusing accuracy of the surface transducer (Xie et al., 2019). Both jointly affect stone fragmentation by altering the stress distribution of shock waves. Based on this, the present study aims to clarify the associations between body composition parameters (including body fat percentage, VAT, and SAT) and ESWL efficacy for PDS, elucidate the core mechanisms by which visceral fat affects lithotripsy efficiency, and provide evidence-based support for formulating individualized treatment regimens. This study fills the research gap regarding “refined body composition parameters and ESWL efficacy for PDS” and establishes a clear distinction from studies on renal calculi by focusing on the unique anatomical and therapeutic requirements of PDS.

## Methods

### Study design and compliance with guidelines

This was a single-center retrospective observational study that followed the Strengthening the Reporting of Observational Studies in Epidemiology (STROBE) guidelines in structuring the study framework. The study was approved by the Medical Ethics Committee of Gongli Hospital, Pudong New Area, Shanghai (ethical approval number: GLYY1s2025-032), and all patients provided written informed consent.

### Study population and inclusion/exclusion criteria

**Study subjects:** A total of 152 consecutive patients with PDS who underwent ESWL at our hospital from January 1, 2021, to January 1, 2025, were initially screened. After exclusions, 147 eligible patients were finally included in the analysis. The five excluded cases consisted of three with missing core data in electronic medical records and two under 18 years of age.

**Inclusion criteria (Definition of complete ESWL success):** Referring to the Guidelines for the Diagnosis and Treatment of Chronic Pancreatitis (2022 Edition) and international efficacy evaluation criteria for ESWL in PDS, complete success was defined as follows: ① radiological cure: thin-slice (≤2 mm) contrast-enhanced CT performed 6–12 weeks after treatment showed no stone fragments with a diameter ≥2 mm in the pancreatic duct system; ② restoration of pancreatic duct morphology: the diameter of the main pancreatic duct ≤3 mm, without dilatation or wall stenosis of the accessory pancreatic ducts; ③ relief of clinical symptoms: a reduction of ≥80% in the Visual Analog Scale score compared with the preoperative level, or complete relief (sustained for ≥3 months); ④ improvement of metabolic function: relief of steatorrhea (fecal elastase > 200 μg/g) and stable blood glucose control (glycated hemoglobin (HbA1c) ≤7%).

**Rationale for including only successfully treated patients:** This study was designed to clarify the quantitative association between body fat parameters and the optimization of ESWL treatment efficacy, rather than identifying risk factors for lithotripsy failure. Thus, focusing on successfully treated cases is supported by a three-fold rationale: First, distinct research focus: risk analysis of failed cases requires using stone non-clearance as the outcome, while this study aims to explore how to adjust ESWL regimens based on body fat parameters to improve success rates. The research hypotheses and outcome indicators of the two approaches are completely independent. Second, control of confounding variables: lithotripsy failure is mostly caused by intrinsic stone characteristics or abnormal equipment parameters. These factors interact with body fat parameters, and analyzing only successfully treated cases can exclude extreme confounding effects from the failure group, enabling more precise localization of the impact of body fat on ”treatment efficiency” (rather than ”success or failure”). Third, clinical value orientation: in clinical practice, clinicians are more in need of clarifying how to optimize regimens by integrating body fat parameters for patients with potential treatment success. Therefore, research focusing on efficacy optimization is more clinically relevant.

### Extracorporeal shock wave lithotripsy procedure

**Diagnostic criteria**: ① Abnormal metabolic indicators: Elevated triglycerides, serum amylase, or glycated hemoglobin (HbA1c) >7%; ② Ultrasonic features: Thickening of the pancreatic parenchyma, dilation of the main pancreatic duct (diameter >3 mm), and stones presenting as hyperechoic masses (diameter >5 mm with posterior acoustic shadowing); ③ CT features: Chronic inflammatory changes of the pancreas, beaded dilation of the main pancreatic duct and its branches, accompanied by intraductal or parenchymal calcification.

**Preoperative evaluation and treatment regimens**: Preoperatively, routine blood tests, coagulation function, hepatic and renal function, and tumor markers (including carcinoembryonic antigen) were performed. Additionally, abdominal contrast-enhanced CT, electrocardiography, and chest radiography were conducted to screen for contraindications. For the treatment, patients were placed in a supine position or a 30° right-decubitus supine position, and stones were localized using a single-beam X-ray rotating C-arm with biplanar imaging.

**ESWL parameter settings**: The energy level was gradually increased from a low level to the target energy levels 4–6 (corresponding to 14–16 kV and 0.62–0.88 mJ/mm^2^), with a shock wave frequency of 60–120 shocks per minute and a maximum of ≤6,000 shocks per treatment session. Fluoroscopic confirmation of positioning accuracy was performed before every 500 shocks and prior to any energy level adjustments, until the stones were fragmented into fragments with a longest diameter <2–3 mm. Serum amylase and lipase were monitored 2–6 h postoperatively, and patients without complications underwent consecutive treatments until the success criteria were met.

### CT image analysis

**Figure 1:**
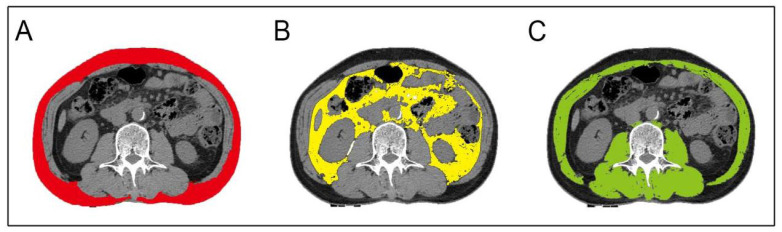
Schematic diagram of different tissue measurements. (A) Subcutaneous adipose tissue (SAT) (red) was calculated by selection of pixels at the L3/4 intervertebral space. (B) Visceral adipose tissue (VAT) (yellow). (C) Skeletal muscle tissue (SMT) (green).

Dual 16-slice CT scanner (Siemens SOMATOM Sensation; Siemens, Munich, Germany) 250 mA whole-abdomen/upper-abdomen CT; tube voltage: 120 kv; data acquisition thickness 5 mm; reconstruction thickness one mm; reconstruction interval one mm. Patients obtained CT scanning within 24 h of admission to hospital. CT of the whole abdomen was performed in 152 patients. 141 patients obtained CT of the upper abdomen. Two consecutive unenhanced CT axial images of the L3/4 vertebral space were retrospectively analyzed by two radiologists who did not have information about the patients. We calculated the cross-sectional areas of fat and muscle, which have been shown to be the best representatives of SAT and VAT, independent of age (Xie et al., 2019). Skeletal muscle areas, including the psoas, paraspinous and abdominal wall muscles were also measured (Fig. 1). Areas of similar signal intensity were outlined and measured based on pixel counts using the Photoshop (Adobe, San Jose, California) “magic wand”. We manually outlined the edges of fat deposits in areas of poorly contrasted adipose tissue, which may be a more accurate way to measure compartment volume. The Hounsfield units (HU) of adipose tissue range from −190∼−30 HU (Aubrey et al., 2014). The mean value of the two images was calculated. Distinguishing between VAT and SAT of the abdominal muscle wall was automatically tracked and manually adjusted (Koda et al., 2007; Gronemeyer et al., 2000). The VAT/SMT ratio was calculated by dividing VAT by SMT.

### Laboratory and patient data collection

Metabolic indicators before and after surgery (including triglycerides, cholesterol, low-density lipoprotein (LDL-C), high-density lipoprotein (HDL-C), HbA1c, transaminases, and amylase) were collected. Demographic characteristics (age, gender, height, weight) and clinical information (smoking history, alcohol consumption history, postoperative complications) were recorded. BMI was calculated as weight (kg) divided by the square of height (m^2^). Body fat percentage was computed using internationally accepted formulas: for males = (1.20 × BMI) + (0.23 × age)−16.2; for females = (1.20 × BMI) + (0.23 × age)−5.4.

### Statistical analysis

IBM SPSS Statistics 26.0 software (IBM Corp. Armonk, NY, USA) was used for comprehensive data analysis. All statistical tests were two-tailed, with the significance level α set at 0.05. A *P*-value < 0.05 was considered statistically significant. The specific statistical methods are as follows:

#### Descriptive statistical methods

Categorical variables: including gender, smoking history, alcohol consumption history, stone location, and other variables, were described as frequencies (percentages) (n (%)) to clearly present the group composition characteristics of the study population.

Continuous variables: including age, BMI, body fat percentage, VAT/SMT ratio, total lithotripsy time, number of lithotripsy sessions, cross-sectional area of stones, and various laboratory indicators, were first tested for distribution type using the Shapiro–Wilk normality test. The test results showed that the Shapiro–Wilk statistic W values of all continuous variables ranged from 0.956 to 0.989, with all *P*-values >0.05, confirming that they all conformed to a normal or approximately normal distribution. Therefore, they were described as “mean ± standard deviation ($\bar {x}$ ± s)” to objectively reflect the central tendency and dispersion of the variables. Total lithotripsy time was defined as the total cumulative fluoroscopic exposure time of each lithotripsy procedure.

#### Correlation analysis between variables

Given that all core variables (exposure variables: BMI, body fat percentage, VAT/SMT ratio; outcome variables: total lithotripsy time, number of lithotripsy sessions) conformed to a normal distribution, the Pearson product-moment correlation test was used to analyze the strength and direction of linear associations between variables. The correlation coefficient r was used to quantify the degree of association, and the *P*-value was used to determine the statistical significance of the correlations, providing a basis for variable selection in subsequent multivariate analysis.

Given that the dependent variable “number of lithotripsy sessions” is a discrete count variable, we further conducted model applicability validation to ensure the reliability of the analysis results: The Shapiro–Wilk test indicated that the distribution of the number of lithotripsy sessions was approximately normal (*W* = 0.972, *P* = 0.103), satisfying the basic assumption of linear regression regarding the distribution of the dependent variable; results of the sensitivity analysis showed that Poisson regression exhibited overdispersion (*α* = 0.876), while the Akaike Information Criterion (AIC) of negative binomial regression (AIC = 578.5) was close to that of linear regression (AIC = 576.2), and the direction and strength of the association between core body composition parameters and outcome variables remained consistent; in addition, the standardized β coefficients of linear regression can directly quantify the impact intensity of each variable, making the interpretation of results more intuitive and consistent with the understanding habits of clinical researchers and readers. Based on the comprehensive results of the aforementioned validation, linear regression was ultimately adopted for the analysis.

#### Multiple linear regression analysis

Hierarchical multiple linear regression models were used to analyze the independent effects of body fat-related parameters and potential confounding variables, with the number of lithotripsy sessions and total lithotripsy time as the dependent variables, respectively. The specific models are as follows:

Model 1: Only BMI, a core exposure variable, was included to analyze its independent predictive effect on the dependent variables.

Model 2: On the basis of Model 1, body fat percentage was added to explore its independent effect and its impact on the predictive effect of BMI.

Model 3: On the basis of Model 2, the VAT/SMT ratio was incorporated to clarify the synergistic predictive effect of the three parameters.

Model 4: All potential confounding variables (gender, age, cross-sectional area of stones, triglycerides, total serum cholesterol, etc.) were included to conduct independent effect analysis after comprehensive adjustment.

In the regression analysis, the standardized regression coefficient (β) was used to reflect the impact intensity of each independent variable on the dependent variables, and the *t*-value and *P*-value were used to verify the statistical significance of the effects. Meanwhile, the variance inflation factor (VIF) was employed to test for multicollinearity. The VIF values of all variables included in the models ranged from 1.04 to 3.31, confirming that no severe multicollinearity existed and ensuring the stability of the model estimation results.

#### Selection criteria for independent variables in regression models

To balance statistical efficiency and clinical rationality while avoiding overfitting, a dual criterion of “priority to clinical rationality and univariate prescreening” was adopted for independent variable selection:

Core exposure variables were directly included based on clinicopathological and physiological mechanisms. The rationale is supported by existing studies confirming that body composition parameters can affect ESWL treatment efficacy by interfering with shock wave transmission efficiency. Additionally, the VAT/SMT ratio, as a key indicator reflecting body composition imbalance, is closely associated with disease severity in patients with chronic pancreatitis, providing a clear basis for clinical relevance.

Potential confounding variables were first subjected to univariate linear regression analysis with the outcome variables as dependent variables, and variables with *P* < 0.15 were selected for inclusion in the multivariate regression model. This threshold setting not only avoids the omission of important confounding variables that may occur with a stringent *P*-value threshold (*e.g.*, *P* < 0.10) but also reduces excessive consumption of model degrees of freedom by irrelevant variables, effectively lowering the risk of model overfitting.

All variables ultimately included in the models underwent clinical significance verification to exclude redundant variables without clear pathophysiological relevance, ensuring the parsimony and interpretability of the models.

## Results

### Baseline characteristics

A total of 147 patients with complete success in ESWL for pancreatic duct stones were finally included in this study, with the following baseline characteristics (Table 1): Gender distribution was predominantly male (109 cases, 74.1%), with 38 females (25.9%), consistent with the clinical epidemiological characteristic of a male predominance in pancreatic duct stones. The mean age was 47.69 ± 14.01 years, covering all adult age groups, indicating good sample representativeness. For core body composition indicators: the mean BMI was 22.14 ± 3.31 kg/m^2^ (within the normal reference range), and the mean body fat percentage was 28.43 ± 10.72%, suggesting significant individual variability and heterogeneity in body fat distribution. The mean VAT/SMT ratio was 0.93 ± 0.45, reflecting a relatively balanced state between visceral fat and skeletal muscle.

**Table 1:** Characteristics of patients. Retrospective collection of 147 patients and the clinical characteristics of these patients are listed.

Variable	Mean (s.d.) or % (n)
Gender	
Male	109 (74.1)
Female	38 (25.9)
Age (years)	47.69 ± 14.01
BMI	22.14 ± 3.31
Body fat percentage (%)	28.43 ± 10.72
VAT/SMT ratio	0.93 ± 0.45
Total lithotripsy time	16.07 ± 9.71
Number of lithotripsy sessions	3.60 ± 1.94
Stone cross-sectional area (cm^2^)	1.88 ± 1.49
Triglycerides	1.57 ± 1.20
Serum total cholesterol	4.05 ± 0.81
Low-density lipoprotein	2.47 ± 0.72
High-density lipoprotein	1.27 ± 0.29
Glycosylated hemoglobin	5.90 ± 1.50
Glutamate transferases	46.51 ± 69.33
Alkaline phosphatase	78.86 ± 51.81
Amylase	109.45 ± 118.96
Smoking	
Yes	47 (32.0)
No	100 (68.0)
Drinking	
Yes	52 (35.4)
No	95 (64.6)
Stone locations	
The head of the pancreas	99 (67.3)
The head of the pancreas, the body of the pancreas, and the tail of the pancreas	42 (29.3)
The pancreatic neck	2 (1.4)
The pancreatic body	3 (2.0)
Total	147 (100)

Regarding treatment outcome indicators: the mean total lithotripsy time was 16.07 ± 9.71 min, and the mean number of lithotripsy sessions was 3.60 ± 1.94, indicating obvious individual differences in ESWL treatment efficiency among different patients. Stone-related characteristics showed that the mean cross-sectional area of stones was 1.88 ± 1.49 cm^2^. The majority of stones were located in the pancreatic head (99 cases, 67.3%), followed by multiple sites involving the pancreatic head + body + tail (42 cases, 28.6%), which is consistent with the anatomical structure of the pancreatic duct and the pathophysiological mechanism of stone formation.

For laboratory indicators: HbA1c was 5.90 ± 1.50% (most patients had well-controlled blood glucose), and the mean amylase was 109.45 ± 118.96 U/L, which was mildly elevated in some patients and may be associated with chronic pancreatic inflammation. In terms of lifestyle: 47 patients (32.0%) had a smoking history, and 52 patients (35.4%) had an alcohol consumption history, both of which are known risk factors for pancreatic duct stones, with distributions consistent with clinical practice.

### Pearson product-moment correlation analysis

Pearson product-moment correlation analysis results showed clear linear associations between core variables, with correlation strengths consistent with clinical logic (Table 2):

**Table 2:** Correlations among study variables. The Pearson correlations (two-tailed) of BMI, Body fat percentage, VAT/SMT, gravel time and number of gravel crushes.

Variable	BMI	Body fat percentage	VAT/SMT	Total lithotripsy time	Number of lithotripsy sessions
BMI	–				
Body fat percentage	0.208^*^	–			
VAT/SMT ratio	0.112	0.373^***^	–		
Total lithotripsy time	0.382^***^	0.544^***^	0.352^***^	–	
Number of lithotripsy sessions	0.354^***^	0.546^***^	0.353^***^	0.929^***^	–

**Notes.**

*** *P* < 0.001.

* *P* < 0.05.

Specifically, BMI was moderately positively correlated with total lithotripsy time (*r* = 0.382, *P* < 0.001) and the number of lithotripsy sessions (*r* = 0.354, *P* < 0.001), indicating that higher BMI was associated with longer lithotripsy time and more sessions. As a traditional surrogate indicator of body fat, BMI’s association with lithotripsy efficiency supports the role of body composition in treatment outcomes. Furthermore, body fat percentage exhibited a strong positive correlation with both total lithotripsy time (*r* = 0.544, *P* < 0.001) and the number of lithotripsy sessions (*r* = 0.546, *P* < 0.001), emerging as the exposure variable with the strongest association with the outcomes. This highlights the most prominent impact of body fat percentage on lithotripsy efficiency. Additionally, the VAT/SMT ratio was moderately positively correlated with total lithotripsy time (*r* = 0.352, *P* < 0.001) and the number of lithotripsy sessions (*r* = 0.353, *P* < 0.001), suggesting that relative visceral fat accumulation impairs lithotripsy efficiency—indicating that the relative distribution of visceral fat *versus* skeletal muscle is also a key determinant of treatment effectiveness.

Notably, total lithotripsy time was very strongly positively correlated with the number of lithotripsy sessions (*r* = 0.929, *P* < 0.001), which aligns with clinical practice: when a single lithotripsy session fails to achieve the target, additional sessions are required, leading to prolonged total time. This high degree of synergy between the two outcome variables validates their reliability. Body fat percentage was moderately positively correlated with the VAT/SMT ratio (*r* = 0.373, *P* < 0.001), while BMI showed a weak and non-statistically significant correlation with the VAT/SMT ratio (*r* = 0.112). These results indicate that body fat percentage is more sensitive to reflecting the balance between visceral fat and skeletal muscle. Importantly, no severe multicollinearity was observed among the three variables (all *r* < 0.8), laying the foundation for subsequent multivariate regression analysis.

The aforementioned correlation strengths are highly consistent with clinical mechanisms: Adipose tissue (especially visceral fat) has a distinct acoustic impedance compared to normal tissues, leading to attenuation of shock wave energy. Higher body fat percentage results in more significant energy loss, necessitating prolonged lithotripsy time or additional sessions to achieve therapeutic goals. As an indirect indicator of body fat, BMI’s moderate correlation further supports the role of body composition in lithotripsy efficiency. Meanwhile, the independent association of the VAT/SMT ratio emphasizes the importance of body fat distribution (rather than total body fat alone), which is consistent with the anatomical characteristic that visceral fat exerts a more pronounced interference on the treatment of retroperitoneal organs. Therefore, the correlation results of this study are not only statistically significant but also aligned with clinicopathological and physiological mechanisms, eliminating the concern of “meaningless low r-values”.

### Relationship between body parameters and number of lithotripsy sessions

Prior to investigating the influencing factors of total lithotripsy time and the number of lithotripsy sessions, a multicollinearity test was performed. The results showed that all VIF values were < 10, indicating no multicollinearity among independent variables. Hierarchical multiple linear regression models were used to analyze the independent effects of various factors on the number of lithotripsy sessions, with the model fit gradually improving (adjusted R^2^: Model 1 = 0.135, Model 2 = 0.302, Model 3 = 0.321, Model 4 = 0.417). The detailed results are presented in Table 3:

**Table 3:** Linear regression analysis of factors associated with number of gravel crushes (*N* = 147).

	Model 1			Model 2			Model 3			Model 4		
	β	*t*	*P*	β	*t*	*P*	β	*t*	*P*	β	*t*	*P*
Constant		−1.15	0.250		−2.52	0.013		−2.87	0.005		−4.06	<0.001
BMI	0.37	4.73	<0.001	0.26	3.75	<0.001	0.25	3.75	<0.001	0.27	3.80	<0.001
Body fat percentage (%)				0.48	6.98	<0.001	0.42	5.71	<0.001	0.38	4.15	<0.001
VAT/SMT ratio							0.17	2.39	0.018	0.20	2.43	0.017
Gender										0.11	1.32	0.188
Age (years)										0.01	0.08	0.940
Stone cross-sectional area (cm^2^)										0.23	3.42	0.001
Triglycerides										0.01	0.12	0.904
Serum total cholesterol										−0.15	−2.08	0.040
Low-density lipoprotein										0.13	1.77	0.080
High-density lipoprotein										0.04	0.52	0.603
Glycosylated hemoglobin										−0.02	−0.25	0.803
Glutamate transferases										0.05	0.78	0.439
Alkaline phosphatase										0.14	2.05	0.042
Amylase										0.01	0.01	0.993
Smoking										−0.02	−0.35	0.729
Drinking										0.07	1.03	0.306
Stone locations										0.09	1.24	0.217

Model 1 (only BMI included): BMI exerted a significantly positive predictive effect on the number of lithotripsy sessions (β = 0.37, *t* = 4.73, *P* < 0.001), indicating that for each 1 kg/m^2^ increase in BMI, the standardized regression coefficient of the number of sessions increased by 0.37. This confirms BMI as a fundamental influencing factor for the number of lithotripsy sessions.

Model 2 (BMI + body fat percentage included): After incorporating body fat percentage into the model, it exhibited a significant predictive effect (β = 0.48, *t* = 6.98, *P* < 0.001) and a stronger predictive power than BMI (β = 0.26, *P* < 0.001). These results demonstrate that the impact of body fat percentage on the number of lithotripsy sessions is independent of BMI, identifying it as a more critical predictor.

Model 3 (BMI + body fat percentage + VAT/SMT ratio included): The VAT/SMT ratio significantly and positively predicted the number of lithotripsy sessions (β = 0.17, *t* = 2.39, *P* = 0.018). Meanwhile, both body fat percentage (β = 0.42, *P* < 0.001) and BMI (β = 0.25, *P* < 0.001) remained statistically significant, suggesting that the three parameters collectively affect the number of lithotripsy sessions without significant mutual interference.

Model 4 (fully adjusted model): After adjusting for 12 potential confounding variables (including gender, age, and cross-sectional area of stones), the effects of core exposure variables remained stably significant: Body fat percentage (β = 0.38, *t* = 4.15, *P* < 0.001), BMI (β = 0.27, *t* = 3.80, *P* < 0.001), and the VAT/SMT ratio (β = 0.20, *t* = 2.43, *P* = 0.017) were all independent positive predictors of the number of lithotripsy sessions; Among confounding variables, cross-sectional area of stones (β = 0.23, *t* = 3.42, *P* = 0.001), total serum cholesterol (β = −0.15, t = −2.08, *P* = 0.040), and alkaline phosphatase (β = 0.14, *t* = 2.05, *P* = 0.042) also showed significant associations. Specifically, larger stone volume and higher alkaline phosphatase levels were associated with more lithotripsy sessions, while elevated total serum cholesterol may have a mild negative correlation with lithotripsy efficiency—the specific mechanism requires further investigation in combination with pancreatic metabolic function; Variables such as gender, age, smoking history, and alcohol consumption history were not statistically significant (*P* > 0.05), indicating that their effects on the number of lithotripsy sessions could be explained by core body fat parameters and stone characteristics.

### Relationship between body parameters and total lithotripsy time

Consistent with the regression analysis of the number of lithotripsy sessions, the hierarchical multiple linear regression models identified the independent effects of various factors on total lithotripsy time. The results were highly consistent with those for the number of lithotripsy sessions (adjusted R^2^: Model 1 = 0.152, Model 2 = 0.305, Model 3 = 0.324, Model 4 = 0.431), verifying the robustness of the conclusions (Table 4):

**Table 4:** Linear regression analysis of factors associated with gravel time (*N* = 147).

	Model 1			Model 2			Model 3			Model 4		
	β	*t*	*P*	β	*t*	*P*	β	*t*	*P*	β	*t*	*P*
Constant		−1.85	0.067		−3.32	0.001		−3.66	<0.001		−4.66	<0.001
BMI	0.39	5.08	<0.001	0.28	4.14	<0.001	0.28	4.14	<0.001	0.32	4.45	<0.001
Body fat percentage (%)				0.48	7.02	<0.001	0.42	5.77	<0.001	0.37	3.94	<0.001
VAT/SMT ratio							0.17	2.33	0.021	0.18	2.13	0.035
Gender										0.07	0.89	0.377
Age (years)										0.05	0.72	0.473
Stone cross-sectional area (cm^2^)										0.15	2.25	0.027
Triglycerides										−0.01	−0.12	0.903
Serum total cholesterol										−0.18	−2.45	0.016
Low-density lipoprotein										0.11	1.53	0.129
High-density lipoprotein										0.09	1.28	0.203
Glycosylated hemoglobin										−0.04	−0.60	0.551
Glutamate transferases										0.08	1.08	0.284
Alkaline phosphatase										0.12	1.77	0.080
Amylase										0.01	0.13	0.894
Smoking										−0.01	−0.05	0.962
Drinking										0.06	0.81	0.423
Stone locations										0.11	1.63	0.105

Progressive effects of Models 1–3: In Model 1, BMI was significantly and positively associated with total lithotripsy time (β = 0.39, *t* = 5.08, *P* < 0.001). After incorporating body fat percentage into Model 2, it exerted the strongest predictive effect (β = 0.48, *t* = 7.02, *P* < 0.001), while the effect of BMI slightly decreased (β = 0.28, *P* < 0.001). In Model 3, the VAT/SMT ratio became statistically significant (β = 0.17, *t* = 2.33, *P* = 0.021). These three parameters synergistically predicted total lithotripsy time, with a trend identical to that observed in the models for the number of lithotripsy sessions.

Model 4 (fully adjusted model): The independent effects of core exposure variables remained stable: body fat percentage (β = 0.37, *t* = 3.94, *P* < 0.001), BMI (β = 0.32, *t* = 4.45, *P* < 0.001), and the VAT/SMT ratio (β = 0.18, *t* = 2.13, *P* = 0.035) all significantly and positively predicted total lithotripsy time. Among confounding variables, cross-sectional area of stones (β = 0.15, *t* = 2.25, *P* = 0.027) and total serum cholesterol (β = −0.18, t = −2.45, *P* = 0.016) showed significant associations, consistent with their effects in the models for the number of lithotripsy sessions. Alkaline phosphatase was marginally significant in this model (*P* = 0.080), which may be attributed to the fact that total lithotripsy time, as a continuous variable, is slightly less sensitive to this indicator than the number of lithotripsy sessions. All other confounding variables were not statistically significant (*P* > 0.05), further supporting the dominant role of core body fat parameters.

## Discussion

Based on clinical data from 147 patients with complete success in ESWL for pancreatic duct stones, this study for the first time identified BMI and the VAT/SMT ratio as independent positive predictors of total lithotripsy time and the number of lithotripsy sessions. Among these parameters, body fat percentage exhibits the strongest association with lithotripsy efficiency, and the independent effect of the VAT/SMT ratio further confirms the critical impact of body fat distribution rather than total body fat alone on treatment efficiency. The determination of this “strongest association” is supported by dual data evidence: in the correlation analysis, the Pearson correlation coefficients of body fat percentage with the number of lithotripsy sessions (*r* = 0.546, *P* < 0.001) and total lithotripsy time (*r* = 0.544, *P* < 0.001) were significantly higher than those of BMI (*r* = 0.354∼0.382, *P* < 0.001) and the VAT/SMT ratio (*r* = 0.352∼0.353, *P* < 0.001), indicating a closer linear association with treatment efficiency; in the multivariate regression analysis, after adjusting for 12 confounding variables, the standardized β coefficients of body fat percentage (β = 0.38 for the number of lithotripsy sessions; β = 0.37 for total lithotripsy time) were still higher than those of BMI (β = 0.27 for the number of lithotripsy sessions; β = 0.32 for total lithotripsy time) and the VAT/SMT ratio (β = 0.20 for the number of lithotripsy sessions; β = 0.18 for total lithotripsy time), reflecting a superior independent impact intensity on the outcome variables.

The lithotripic efficacy of shock waves relies on energy transmission efficiency and focusing accuracy, and body fat distribution interferes with shock wave propagation and focusing at the physical level. Adipose tissue impairs ESWL outcomes for pancreatic duct stones through dual physical mechanisms: adipose tissue has a significantly different acoustic impedance from muscle tissue, leading to refraction and scattering of shock waves at the fat-muscle interface, with energy loss increasing exponentially with fat thickness (Mast et al., 1998; Che et al., 2021). The strong correlation between body fat percentage and lithotripsy outcomes in this study corroborates this mechanism. Located retroperitoneally, the pancreatic duct is directly affected by thickened SAT that prolongs the shock wave transmission path, while the fat pad formed by VAT around the pancreas further exacerbates energy attenuation—particularly the pancreatic head, which is surrounded by more visceral fat, resulting in more pronounced loss of focused energy. The uneven distribution of visceral fat alters the propagation phase of shock waves, increasing focal shift, which also explains why the VAT/SMT ratio independently predicts lithotripsy efficiency (β = 0.18∼0.20, *P* < 0.05) with a stronger effect than BMI alone.

At the metabolic level, as a metabolically active endocrine organ, visceral fat affects the pancreatic microenvironment by secreting inflammatory factors (*e.g.*, TNF-α, IL-6), which may indirectly alter the physical properties of stones (Alexaki, 2024). The association between metabolism-related indicators and lithotripsy outcomes in this study further supports this mechanism: serum total cholesterol exhibits a significant negative correlation with both total lithotripsy time (β = −0.18, *P* = 0.016) and the number of lithotripsy sessions (β = −0.15, *P* = 0.040), suggesting that it may exert a mild impact on the shock wave sensitivity of stones by participating in the regulation of lipid metabolism; alkaline phosphatase is only significantly associated with the number of lithotripsy sessions (β = 0.14, *P* = 0.042) and has no statistical association with total lithotripsy time (*P* = 0.080). As a metabolism-related indicator linked to lithotripsy efficiency, it is hypothesized that alkaline phosphatase may indirectly increase the requirement for lithotripsy sessions by affecting the degree of stone calcification or the pancreatic tissue microenvironment. These results indicate that body fat accumulation may interfere with the body’s metabolic processes, alter the physical properties of stones (*e.g.*, calcification degree, hardness) or the local pancreatic environment, and further reduce the fragmentation effect of shock waves on stones. This mechanism is consistent with findings in kidney stone research, but pancreatic duct stones are mostly secondary to chronic pancreatitis—adipokine regulation of pancreatic fibrosis and pancreatic juice composition may exert a synergistic effect, which requires verification in subsequent studies (Ye et al., 2025).

Our findings align with relevant studies on kidney stone ESWL but also exhibit fundamental differences. Both confirm a negative correlation between body fat parameters and lithotripsy efficiency, with core mechanisms involving energy attenuation and focusing interference caused by adipose tissue. However, three key distinctions exist: first, skin-to-stone distance (SSD) is a core indicator in kidney stone ESWL, but due to the deeper anatomical location of pancreatic duct stones, the VAT/SMT ratio has a more significant impact (*r* = 0.352∼0.353, *P* < 0.001) in the current study, while SSD measurement has poor repeatability and limited clinical value (Pareek et al., 2005; Gonulalan et al., 2014). Second, kidney stones are predominantly composed of calcium oxalate (high hardness), whereas pancreatic duct stones are mostly complexes of calcium carbonate and proteins (uneven calcification), making the energy attenuation caused by body fat more impactful on pancreatic duct stones, which require longer time to reach the lithotripic threshold (Asplin et al., 1998; Bockman et al., 1986; Hammad & Balakrishnan, 2010). Finally, the pancreas is adjacent to vital organs, and obese patients cannot compensate for fat interference by simply increasing energy output—instead, they rely on additional sessions, which differs from the strategy of moderately increasing energy density for kidney stones (Hammad & Balakrishnan, 2010; Yokoyama et al., 2022).

Most existing ESWL studies on pancreatic duct stones have focused on factors such as stone size and CT value (cross-sectional area of stones in this study: β = 0.15∼0.23, *P* < 0.05), while the impact of body fat parameters has long been overlooked. Although many studies have linked BMI to the progression of chronic pancreatitis, none have explored its role in treatment efficiency. The innovation of this study lies in the first-time inclusion of refined body fat parameters, confirming their predictive value independent of BMI. Through hierarchical regression models, confounding factors such as gender, age, and metabolic indicators were excluded, clarifying the independent effects of body fat parameters. Focusing on successful cases avoided the extreme confounding effects of failed groups (*e.g.*, stone impaction, equipment abnormalities), enabling more accurate quantification of the association between body fat and treatment efficiency.

Based on these findings, clinical practice can optimize ESWL protocols for pancreatic duct stones as follows: it is recommended to measure the VAT/SMT ratio at the L3/4 intervertebral space *via* CT; when the ratio >1.5, the number of lithotripsy sessions may increase by ≥ 2, and patients should be informed in advance to adjust treatment expectations. For patients with a body fat percentage >35%, the feasibility of combined endoscopic therapy should be prioritized to avoid pancreatic injury caused by repeated ESWL. For obese patients, a “low energy, high frequency” strategy can be adopted, with shortened intervals for fluoroscopic positioning to balance lithotripsy efficiency and safety. Currently, there are no clear body fat-related exclusion criteria for ESWL in pancreatic duct stones; based on this study, it is suggested that VAT/SMT >2.0 combined with a stone cross-sectional area >three cm^2^ be classified as a relative contraindication. ESWL is extremely inefficient in such patients, and surgical treatment should be directly recommended.

This study has several limitations: it adopts a single-center retrospective design with a limited sample size, including only successful cases, which may introduce selection bias and prevent analysis of the association between body fat parameters and lithotripsy failure. Total lithotripsy time may be influenced by the surgeon’s proficiency. Differences in parameters of various lithotripters (*e.g.*, shock wave waveform, focusing mode) were not recorded, which may have potential confounding effects on the results. Molecular indicators such as adipokines (*e.g.*, leptin, adiponectin) were not included, precluding in-depth exploration of metabolic mechanisms. Future studies should conduct multi-center prospective research, include failed cases, extend follow-up periods, and combine three-dimensional imaging reconstruction technology to further verify the causal association between body fat distribution and ESWL efficacy. It should be noted that the core purpose of this study was to explore the association pattern between refined body composition parameters and lithotripsy efficiency, and no formal model comparison analyses such as variance decomposition or AIC comparison were conducted. Therefore, no absolute conclusion that “body fat percentage has the optimal predictive power” was drawn, and only its relative advantage in association strength was confirmed. Future studies can further quantify the predictive efficacy of each body composition parameter by constructing competing prediction models and conducting external validation, so as to provide more precise evidence-based support for clinical efficacy prediction.

## Conclusion

Body fat percentage, BMI, and the VAT/SMT ratio are independent predictors of ESWL treatment efficiency for pancreatic duct stones, which affect lithotripsy outcomes through dual mechanisms of physical obstruction and metabolic regulation. In clinical practice, individualized adjustment of treatment protocols should be performed by incorporating CT-derived body fat parameters to improve treatment efficiency and reduce the risk of complications.

## Supplemental Information

10.7717/peerj.21112/supp-1 Supplemental Information 1 Patient clinical data aggregation

10.7717/peerj.21112/supp-2 Supplemental Information 2 STROBE checklist

## Abbreviations

VAT: Visceral adipose tissue
SAT: subcutaneous adipose tissue
SMT: skeletal muscle tissue
CT: computed tomography
CP: Chronic pancreatitis
ESWL: Extracorporeal shock wave lithotripsy
BMI: Body Mass Index
VAS: visual analog score
LDL: Low-Density Lipoprotein
HDL: High-Density Lipoprotein
HU: Hounsfield units
SSD: skin-to-stone distance
PDS: pancreatic duct stones

## Additional Information and Declarations

### Data Availability

The following information was supplied regarding data availability: The raw data is available in the Supplemental File and at Figshare: - ning, weichen (2025). Impact of Body Fat Content on Extracorporeal Shock Wave Lithotripsy for Pancreatic Duct Stones: A Retrospective Cohort Study. figshare. Dataset. https://doi.org/10.6084/m9.figshare.30940517.v1.
