# Supplementary material for: Impact of body fat content on extracorporeal shock wave lithotripsy for pancreatic duct stones: a retrospective cohort study
Source: PeerJ. 2026 Apr 9;14:e21112. doi: 10.7717/peerj.21112 (PMC13070325; doi:10.7717/peerj.21112)
Supplement: Supplemental Information 2 [file peerj-14-21112-s002.doc]

STROBE Statement—checklist of items that should be included in reports of observational studies

|  | Item No. | Recommendation | Page  No. | Relevant text from manuscript |
| --- | --- | --- | --- | --- |
| **Title and abstract** | 1 | (*a*) Indicate the study’s design with a commonly used term in the title or the abstract | 1-3 | Impact of Body Fat Content on Extracorporeal Shock Wave Lithotripsy for Pancreatic Duct Stones: A Retrospective Cohort Study |
|  |  | (*b*) Provide in the abstract an informative and balanced summary of what was done and what was found | 1-45 | BMI, percent body fat, and VAT/SMT were positively correlated with lithotripsy time and the number of complete lithotripsy removals, and body fat content negatively affected the effectiveness of Extracorporeal Shock-Wave Lithotripsy (ESWL). |
| Introduction | | | |  |
| Background/rationale | 2 | Explain the scientific background and rationale for the investigation being reported | 47-107 | Obesity has been shown to be an independent risk factor for the progression of chronic pancreatitis and significantly increases the incidence of systemic complications and all-cause mortality |
| Objectives | 3 | State specific objectives, including any prespecified hypotheses | 108-118 | the aim of this study was to focus on the mechanism of visceral fat deposition on stone fragmentation efficiency by analyzing the correlation between treatment outcomes and body composition parameters in patients with pancreatic ductal stones who underwent extracorporeal shockwave lithotripsy, with the aim of providing a new evidence-based rationale for the individualized treatment of such patients. |
| Methods | | | |  |
| Study design | 4 | Present key elements of study design early in the paper | 142-153 | laboratory tests for metabolic parameters |
| Setting | 5 | Describe the setting, locations, and relevant dates, including periods of recruitment, exposure, follow-up, and data collection | 121-126 | We selected a total of 152 consecutive patients with complete removal of pancreatic ductal stones by extracorporeal shock wave lithotripsy who were hospitalized at Gongli Hospital |
| Participants | 6 | (*a*) *Cohort study*—Give the eligibility criteria, and the sources and methods of selection of participants. Describe methods of follow-up  *Case-control study*—Give the eligibility criteria, and the sources and methods of case ascertainment and control selection. Give the rationale for the choice of cases and controls  *Cross-sectional study*—Give the eligibility criteria, and the sources and methods of selection of participants | 120-139 | The selection criteria were no residual stones on postoperative CT examination: no visible stone fragments (<2 mm in diameter) in the pancreatic ductal system on thin-layer (≤2 mm) enhanced CT scans performed 6-12 weeks after treatment. |
|  |  | (*b*) *Cohort study*—For matched studies, give matching criteria and number of exposed and unexposed  *Case-control study*—For matched studies, give matching criteria and the number of controls per case | 120-139 |  |
| Variables | 7 | Clearly define all outcomes, exposures, predictors, potential confounders, and effect modifiers. Give diagnostic criteria, if applicable | 141-188 | Diagnostic criteria，Preoperative evaluation |
| Data sources/ measurement | 8* | For each variable of interest, give sources of data and details of methods of assessment (measurement). Describe comparability of assessment methods if there is more than one group | *189-207* |  |
| Bias | 9 | Describe any efforts to address potential sources of bias | 190-207 | Areas of similar signal intensity were outlined and measured based on pixel counts using the Photoshop “magic wand”. |
| Study size | 10 | Explain how the study size was arrived at | 135-139 | In these samples, we excluded participants with missing electronic medical record data and under 18 years of age. |

Continued on next page

| Quantitative variables | 11 | Explain how quantitative variables were handled in the analyses. If applicable, describe which groupings were chosen and why | 224-230 | We used IBM SPSS (version 26.0) for analysis. Sociodemographics, clinical laboratory data, and key variable characteristics were analyzed using frequencies, percentages, means, and standard errors. |
| --- | --- | --- | --- | --- |
| Statistical methods | 12 | (*a*) Describe all statistical methods, including those used to control for confounding | 224-230 | Standardized regression coefficients were calculated using linear regression models for time to lithotripsy and number of complete lithotripsy removals for BMI, body fat percentage, and VAT/SMT, respectively. alpha test level was < 0.05, and the difference was statistically significant. |
|  |  | (*b*) Describe any methods used to examine subgroups and interactions |  |  |
|  |  | (*c*) Explain how missing data were addressed |  |  |
|  |  | (*d*) *Cohort study*—If applicable, explain how loss to follow-up was addressed  *Case-control study*—If applicable, explain how matching of cases and controls was addressed  *Cross-sectional study*—If applicable, describe analytical methods taking account of sampling strategy |  |  |
|  |  | (*e*) Describe any sensitivity analyses |  |  |
| Results | | | | |
| Participants | 13* | (a) Report numbers of individuals at each stage of study—eg numbers potentially eligible, examined for eligibility, confirmed eligible, included in the study, completing follow-up, and analysed | 233-253 | We retrospectively collected 147 patients. The clinical characteristics of these patients are listed in Tables 1. |
|  |  | (b) Give reasons for non-participation at each stage |  |  |
|  |  | (c) Consider use of a flow diagram |  |  |
| Descriptive data | 14* | (a) Give characteristics of study participants (eg demographic, clinical, social) and information on exposures and potential confounders | 233-253 | Of these, 109 were males and 38 were females. In terms of drinking, 52 people drank alcohol, accounting for 35.4%, and 95 people did not drink alcohol, accounting for 64.6%. |
|  |  | (b) Indicate number of participants with missing data for each variable of interest |  |  |
|  |  | (c) *Cohort study*—Summarise follow-up time (eg, average and total amount) |  |  |
| Outcome data | 15* | *Cohort study*—Report numbers of outcome events or summary measures over time | *255-262* | *As shown in Table 2 presenting the Pearson correlations (two-tailed) of BMI, Body fat percentage, VAT/SMT, gravel time and number of gravel crushes.* |
|  |  | *Case-control study—*Report numbers in each exposure category, or summary measures of exposure |  |  |
|  |  | *Cross-sectional study—*Report numbers of outcome events or summary measures |  |  |
| Main results | 16 | (*a*) Give unadjusted estimates and, if applicable, confounder-adjusted estimates and their precision (eg, 95% confidence interval). Make clear which confounders were adjusted for and why they were included | 263-269 | Table 3 displays the linear regression results of the factors associated with the number of gravel crushes. |
|  |  | (*b*) Report category boundaries when continuous variables were categorized | 270-276 | Table 4 displays the linear regression results of the factors associated with the gravel time. |
|  |  | (*c*) If relevant, consider translating estimates of relative risk into absolute risk for a meaningful time period |  |  |

Continued on next page

| Other analyses | 17 | Report other analyses done—eg analyses of subgroups and interactions, and sensitivity analyses |  |  |
| --- | --- | --- | --- | --- |
| Discussion | | | | |
| Key results | 18 | Summarise key results with reference to study objectives | 279-309 | The efficiency of ESWL, the first-line treatment for stones, is influenced by several factors, among which the body fat ratio and visceral fat content play a key role through multiple mechanisms such as energy attenuation, stone localization, and metabolic microenvironment. |
| Limitations | 19 | Discuss limitations of the study, taking into account sources of potential bias or imprecision. Discuss both direction and magnitude of any potential bias | 279-309 | In clinical practice, patients' body fat distribution, metabolic status, and stone characteristics should be evaluated comprehensively to adopt individualized energy regimens and device selection. |
| Interpretation | 20 | Give a cautious overall interpretation of results considering objectives, limitations, multiplicity of analyses, results from similar studies, and other relevant evidence | 310-321 | Elevated body fat ratio significantly reduces the success rate of ESWL. Meta-analysis showed that patients with a BMI ≥30 kg/m² had a 1.9 times higher risk of ESWL failure than normal-weight individuals and a 25-30% reduction in stone clearance |
| Generalisability | 21 | Discuss the generalisability (external validity) of the study results | 322-340 |  |
| Other information | |  | | |
| Funding | 22 | Give the source of funding and the role of the funders for the present study and, if applicable, for the original study on which the present article is based | 361-364 | This study was supported by The Investigator-initiated Trial Program of Shanghai Pudong New Area Health Commission (the Medical and Industrial Integration Program) (No.2025-PWYC-04). |

*Give information separately for cases and controls in case-control studies and, if applicable, for exposed and unexposed groups in cohort and cross-sectional studies.

**Note:** An Explanation and Elaboration article discusses each checklist item and gives methodological background and published examples of transparent reporting. The STROBE checklist is best used in conjunction with this article (freely available on the Web sites of PLoS Medicine at http://www.plosmedicine.org/, Annals of Internal Medicine at http://www.annals.org/, and Epidemiology at http://www.epidem.com/). Information on the STROBE Initiative is available at www.strobe-statement.org.
